# Acute toxicity of the fungicide captan to honey bees and mixed evidence for synergism with the insecticide thiamethoxam

**DOI:** 10.1038/s41598-024-66248-x

**Published:** 2024-07-08

**Authors:** Daiana De Souza, Christine Urbanowicz, Wee Hao Ng, Nicolas Baert, Ashley A. Fersch, Michael L. Smith, Scott H. McArt

**Affiliations:** 1 Department of Entomology, Cornell University, Ithaca, NY 14853 USA; 2 Department of Biological Sciences, Auburn University, Auburn, AL 36849 USA; 3 Department of Collective Behaviour, Max Planck Institute of Animal Behavior, 78464 Konstanz, Germany

**Keywords:** *Apis mellifera*, Ecotoxicology, Synergism, Antagonism, In vitro rearing, Ecology, Environmental sciences

## Abstract

Honey bees are commonly co-exposed to pesticides during crop pollination, including the fungicide captan and neonicotinoid insecticide thiamethoxam. We assessed the impact of exposure to these two pesticides individually and in combination, at a range of field-realistic doses. In laboratory assays, mortality of larvae treated with captan was 80–90% greater than controls, dose-independent, and similar to mortality from the lowest dose of thiamethoxam. There was evidence of synergism (i.e., a non-additive response) from captan-thiamethoxam co-exposure at the highest dose of thiamethoxam, but not at lower doses. In the field, we exposed whole colonies to the lowest doses used in the laboratory. Exposure to captan and thiamethoxam individually and in combination resulted in minimal impacts on population growth or colony mortality, and there was no evidence of synergism or antagonism. These results suggest captan and thiamethoxam are each acutely toxic to immature honey bees, but whole colonies can potentially compensate for detrimental effects, at least at the low doses used in our field trial, or that methodological differences of the field experiment impacted results (e.g., dilution of treatments with natural pollen). If compensation occurred, further work is needed to assess how it occurred, potentially via increased queen egg laying, and whether short-term compensation leads to long-term costs. Further work is also needed for other crop pollinators that lack the social detoxification capabilities of honey bee colonies and may be less resilient to pesticides.

## Introduction

The western honey bee, *Apis mellifera*, is the most important managed pollinator in the world, contributing approximately half of crop pollination services worldwide^1^. Unfortunately, in recent years high rates of honey bee colony losses have been observed^2–6^. Lack of floral resources, exposure to pesticides, and pathogens/parasites are among the major stressors contributing to high colony loss rates and weakened pollinator populations^7^. Furthermore, pesticide residues in pollen and nectar collected by bees are often found at levels known to influence honey bee susceptibility to parasites and pathogens^8–10^, foraging behaviors^11,12^, and growth and survival^11,13–15^.

During crop pollination, honey bees are commonly co-exposed to multiple pesticides^16–19^. Fungicide residues are often the most abundant pesticides found in bee-collected pollen, bee bread, and other hive products^16,18–24^. This should not be surprising since for most fungicides, applications can be used during crop bloom, when honey bees and other pollinators are actively foraging at flowers. But honey bees are also exposed to insecticides and other pesticides during crop pollination. For example, honey bees conducting blueberry pollination in Michigan were simultaneously exposed to an average of 35 pesticides in bee-collected pollen^17^, with the majority of risk coming from the organophosphate insecticide chlorpyrifos (max concentration in pollen = 214 parts per billion; ppb), and the neonicotinoid insecticides clothianidin (max concentration = 35 ppb), imidacloprid (max concentration = 9 ppb), and thiamethoxam (max concentration = 15 ppb^25^). Honey bees conducting apple pollination in New York were also commonly exposed to insecticides; freshly collected beebread from hives at 24 of 30 orchards contained insecticides, with the majority of risk attributed to the oxadiazine insecticide indoxacarb (max concentration in pollen = 918 ppb) and the neonicotinoid insecticide thiamethoxam (max concentration in pollen = 48 ppb^18^).

Neonicotinoids contribute to pesticide risk during crop pollination; a worldwide review of pesticide risk to bees found that the neonicotinoid thiamethoxam was one of the three highest-risk pesticides to honey bees^24,26^. Thiamethoxam is a systemic and environmentally persistent insecticide, which increases the likelihood of exposure via pollen, nectar, plant guttation fluids, soils, and other environmental matrices for days, months, or even years^27–29^. Thiamethoxam’s action as a neurotoxin can lead to paralysis and death of adult bees by binding to nicotinic acetylcholine receptors, and at sublethal exposure levels, can affect the ability of bees to perform important tasks inside and outside of the hive^27,30–32^. Exposure of larvae to thiamethoxam is also known to affect survival and physiology of honey bee postembryonic stages^33–35^.

Although fungicide exposure is generally considered safe for bees, concern has recently been raised about the risk posed from some fungicides, including captan^36–38^. Captan is one of the most widely used fungicides in the United States and the most-sprayed fungicide during apple bloom in New York State^18^. Officially, captan is considered relatively non-toxic to adult honey bees^39,40^. However, one study found that 100% of honey bee larvae died following exposure to field-realistic doses of Captan^41^, though this study used the pesticide product Captan, which contains the active ingredient captan as well as other ingredients and therefore it’s impossible to know whether the observed effects were due solely to captan or other co-formulants. Fungicides can also have indirect negative consequences on bees, including via interactions with pathogens/parasites^9,19,42^, disruption of bee microbiota^43,44^, and synergistic interactions with insecticides, including synergisms with the neonicotinoid thiamethoxam^24,45–47^. However, little is known regarding interactions between captan and any insecticides, including thiamethoxam. Such information is important since some fungicide-insecticide combinations lead to synergisms and some do not (e.g., Iverson et al.^45^), and synergisms are also known to be dose-dependent^48^, which can be useful in guiding recommendations regarding which fungicides are safest to apply during crop pollination.

Here, we investigate the individual and interactive effects of two commonly used pesticides, the neonicotinoid insecticide thiamethoxam and the fungicide captan, on honey bee larval development and full-colony growth and survival. In the lab, we exposed larvae to three field-realistic doses of each pesticide individually and in combination, monitoring survival and development. In the field, we exposed full colonies to each pesticide individually and in combination at the lowest dose used in the laboratory assays, monitoring colony performance and survival over a full calendar year. This study addresses three main questions:

1. What are the direct effects of captan and thiamethoxam on development and survival of individual larvae and full colonies?
2. Does captan-thiamethoxam co-exposure synergistically or antagonistically affect development and survival of individual larvae and full colonies?
3. Do in vitro assays with individual larvae scale up to predict colony-level outcomes?

## Results

### In vitro laboratory assays

**Figure 1:**
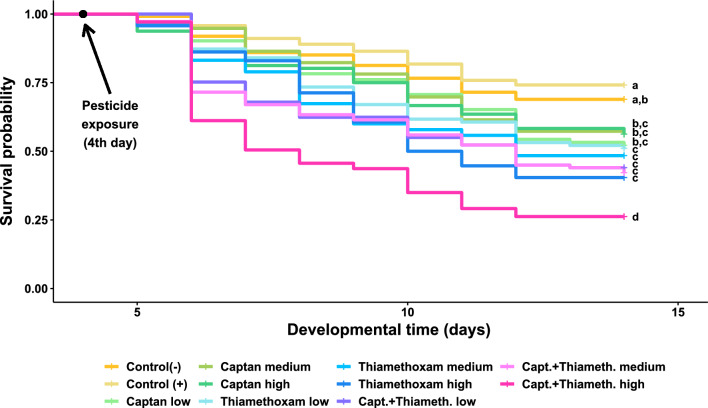
Survival of honey bee larvae exposed to different doses of captan, thiamethoxam, or captan + thiamethoxam. The exposure scenarios include: liquid diet with no additional chemicals (Control (−), *n* = 235), exposure to the solvent acetone (Control (+), *n* = 236), exposure to captan at concentrations of 0.10 ng/ul (Captan low, *n* = 92), 0.50 ng/ul (Captan medium, *n* = 96) and 2.0 ng/ul (Captan high, *n* = 96); exposure to thiamethoxam at concentrations of 0.01 ng/ul (Thiamethoxam low, *n* = 94), 0.07 ng/ul (Thiamethoxam medium, *n* = 95) and 1.44 ng/ul (Thiamethoxam high, *n* = 94); and exposure to a combination of captan at a fixed concentration of 2.0 ng/ul blended with thiamethoxam at concentrations of 0.01 ng/ul (Capt. + Thiameth. low, *n* = 109), 0.07 ng/ul (Capt. + Thiameth. medium, *n* = 109) and 1.44 ng/ul (Capt. + Thiameth. high, *n* = 103). Pesticide exposure started on Day 4 of larval development according to recommended protocols (Schmehl et al. 2016, OECD 2016). Significant pairwise differences in overall survival using Tukey’s post-hoc contrasts (*p* < 0.05) are indicated using compact letter displays.

**Figure 2:**
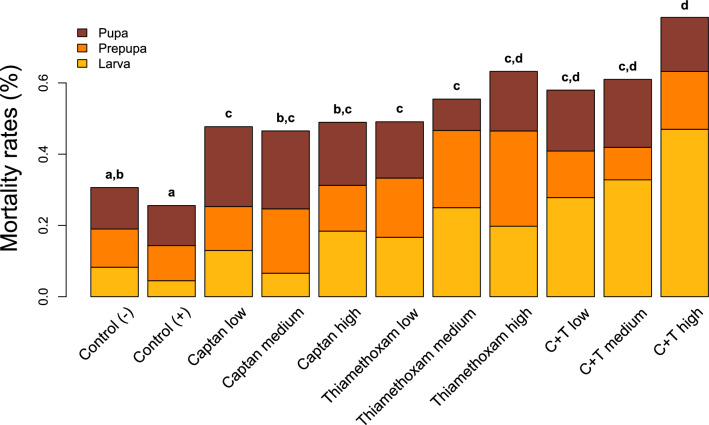
Mortality rates by developmental stage of honey bees exposed to different doses of captan, thiamethoxam, or captan + thiamethoxam. Developmental stages: larva (until larvae finished eating the provided diet and assumed a vertical position, generally encompassing days 1–6), prepupa (larvae in pre-pupation stage, generally occurring during days 7–9) and pupa (the last ontogenetic stage of development, generally occurring between day 10–14). Significant differences in overall survival between treatment groups (*p* < 0.05) are indicated using compact letter displays.

We assessed the impact of pesticide treatment on survival of immature bees in two ways: first using a Cox proportional hazards model for survivorship (Fig. 1), and second using a logistic model for the percentage mortality at the end of the trial (Fig. 2). Survival of immature bees in the positive and negative control groups (with and without acetone used as a solvent) were 74% and 69%, respectively, and were not significantly different in either model (Cox model *p*_Tukey_ = 0.95; logistic model *p*_Tukey_ = 0.97). All pesticide treatments resulted in significantly greater mortality than the positive control (i.e., the acetone-treated control; Tables S2 and S3). Treatment of larvae with the fungicide captan resulted in an 80–90% increase in mortality compared to the positive controls, with no significant difference between dosages (Fig. 2, Tables S2 and S3). Treatment of larvae with the insecticide thiamethoxam at the low, medium and high dosages increased mortality rates by 90%, 120% and 150%, respectively, compared to the positive control, although again the differences between dosages were not significant (Fig. 2, Tables S2 and S3). Finally, the combination of captan (highest dosage) with thiamethoxam at the low, medium and high dosages demonstrated 130%, 140% and 210% increase in mortality rates, respectively, compared to the positive control (Fig. 2). Mortality from the combination of captan with the high dose of thiamethoxam was significantly greater than mortality from the combination with the medium or low doses via the Cox model (Table S2), but these differences were not observed via the logistic model (Table S3).

We developed an improved method to assess interactions between pesticides that identifies non-additive responses (synergism or antagonism) using a log-binomial generalized linear model (GLM) for survivorship. We did not detect any significant interactions on overall survivorship (see Table S4). However, when survivorship within each developmental stage was analyzed separately, we detected statistically significant synergistic interactions (leading to higher-than-expected mortality) at the larval stage in the bioassay when captan was crossed with thiamethoxam at highest dose (LR = 7.932, *p*_Holm_ = 0.044). For other developmental stages and chemical dosages, no significant interactions were observed (see Table S4).

### Field trials with whole colonies

**Figure 3:**
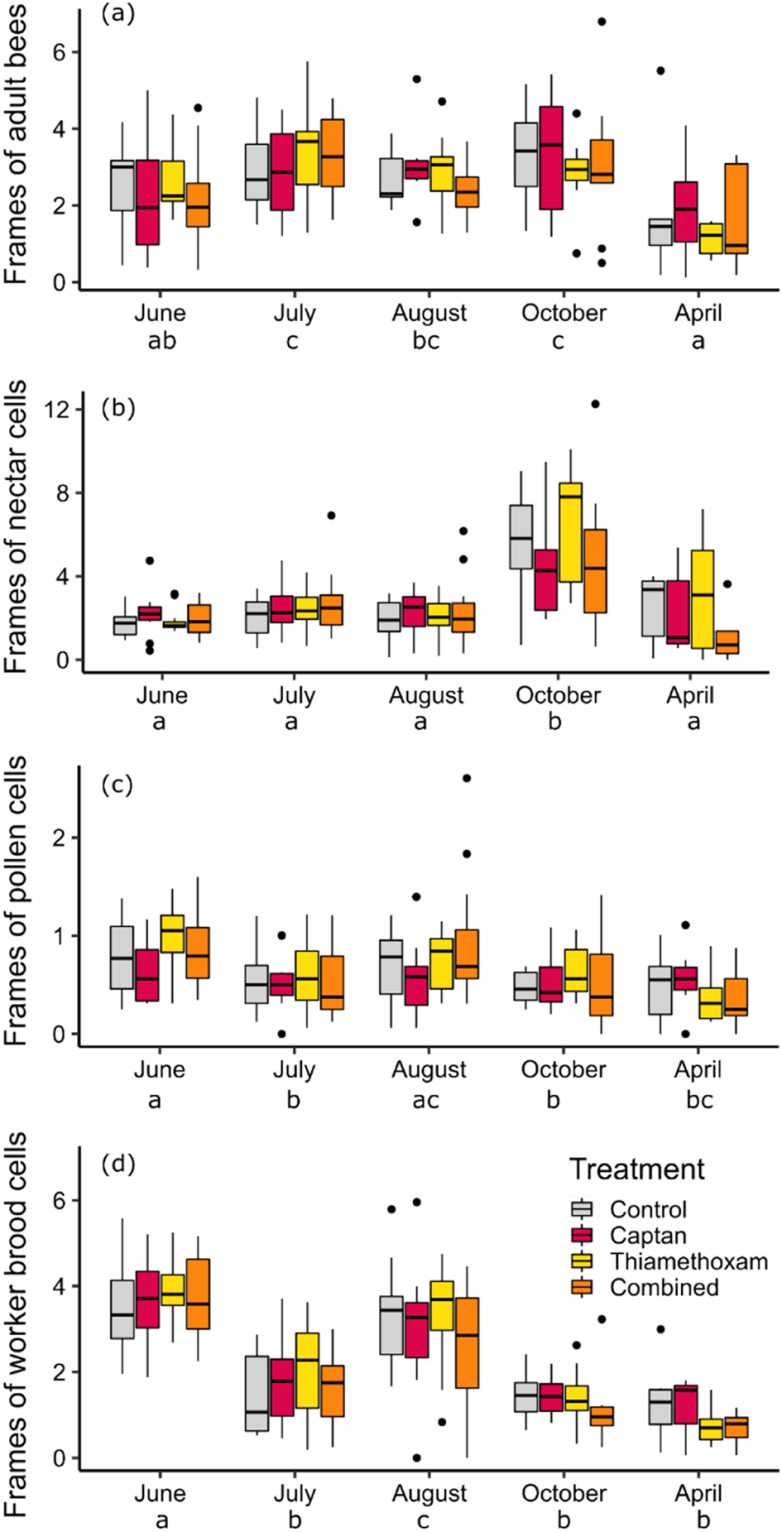
Number of frames covered by adult bees (**a**), nectar cells (**b**), pollen cells (**c**), and worker brood cells (**d**) among the three pesticide treatments and control colonies. The proportion of each frame covered by adult bees, nectar cells, pollen cells and brood cells was visually estimated, and these proportions were summed across all frames in a hive. Different letters below month names indicate significant differences between months (*p* < 0.05) according to post-hoc Tukey pairwise comparisons.

We assessed the impact of pesticide treatment using nine performance metrics for all colonies over a full year – number of adult bees, nectar cells, pollen cells, worker brood cells, drone brood cells, varroa levels, chalkbrood presence, queen supersedure events, and colony survival. No significant difference was observed among treatment groups and there was no treatment × month interaction for number of adult bees, nectar cells, pollen cells, worker brood cells, or drone brood cells, but each of these performance metrics varied significantly among months (Table 1, Fig. 3; Supplemental material Fig. S1).

**Table 1:** Likelihood ratio test results for separate models to test the effect of pesticide treatment on five performance metrics–area of adult bees, nectar cells, pollen cells, worker brood cells, and drone brood cells.

Response	Source	Degrees of freedom	χ^2^	*p*
Adult bees	Treatment	3	0.92	0.82
	Month	4	39.99	< 0.0001
	Bee yard	2	0.47	0.79
	Treatment × month	12	10.61	0.56
Nectar cells	Treatment	3	0.60	0.90
	Month	4	97.57	< 0.0001
	Area of adult bees	1	3.43	0.064
	Bee yard	2	1.99	0.37
	Treatment × month	12	17.15	0.14
Pollen cells	Treatment	3	3.05	0.38
	Month	4	26.22	< 0.0001
	Area of adult bees	1	1.40	0.24
	Bee yard	2	2.61	0.27
	Treatment × month	12	13.31	0.35
Brood cells	Treatment	3	1.81	0.61
	Month	4	159.27	< 0.0001
	Area of adult bees	1	18.33	< 0.0001
	Bee yard	2	0.40	0.82
	Treatment × month	12	6.52	0.89
Drone brood cells	Treatment	3	3.66	0.30
	Month	4	62.77	< 0.0001
	Area of adult bees	1	31.97	< 0.0001
	Bee yard	2	7.21	0.027
	Treatment × month	12	5.47	0.94

Consumption of pollen patties containing the treatments (captan, thiamethoxam, captan + thiamethoxam, or control) did not vary by treatment, apiary location, or the treatment × month interaction (Supplemental material Table S5), but consumption varied significantly among months and was positively correlated with the area of adult bees in a colony (β = 4.35 ± 0.06; Table S5, Fig. S2).

**Figure 4:**
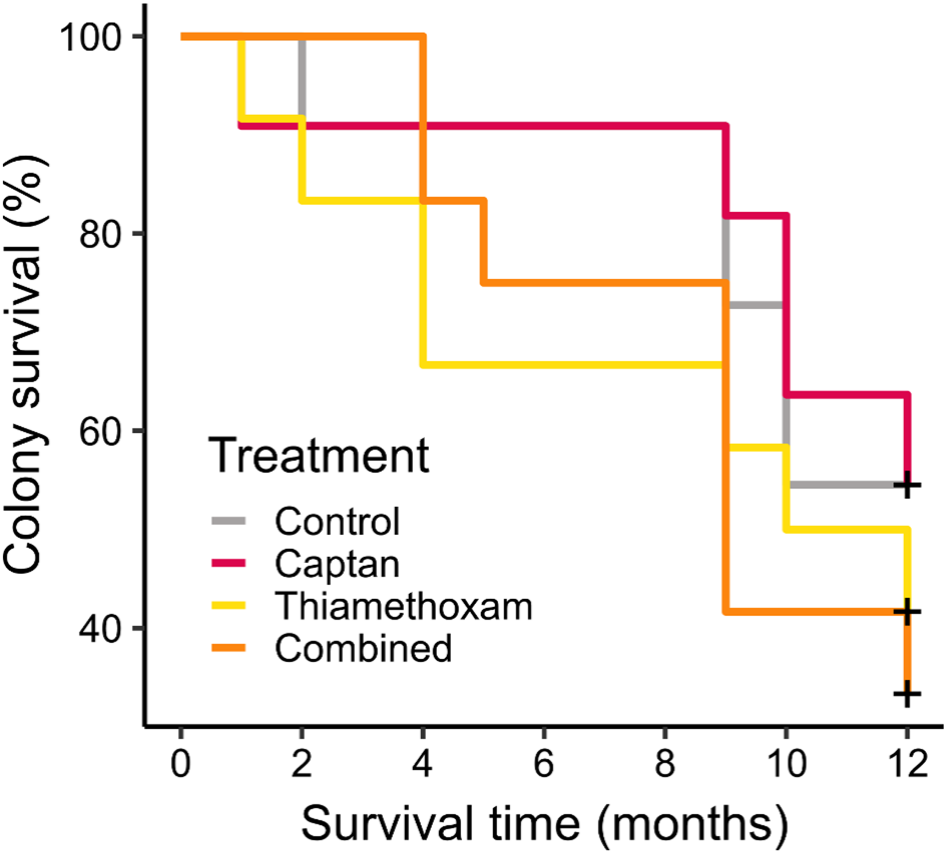
Survival curves for the three pesticide treatments and control colonies between June 2016 and June 2017. “+” indicates censored observations (i.e., percent of colonies surviving at 12 months, when experiment ended).

Queen supersedure occurred in eleven of the 46 colonies, but supersedure was not related to treatment (χ^2^_3_ = 6.43, *p* = 0.093). Varroa levels varied significantly among months, with levels higher in September 2016 than in July 2016 or April 2017 (Supplemental material Table S6; Fig. S3), but did not vary by treatment. Chalkbrood was present in 25 of the 46 hives. The presence of chalkbrood at any point in the study was not related to treatment (χ^2^_3_ = 2.15, *p* = 0.54) or apiary location (χ^2^_3_ = 2.26, *p* = 0.32). Finally, no significant difference in colony survival was observed among treatments (χ^2^_11_ = 10.4, *p* = 0.51, Fig. 4).

## Discussion

Our data indicate that exposure of honey bee larvae to field-realistic levels of the fungicide captan, neonicotinoid insecticide thiamethoxam, and the combination of captan + thiamethoxam significantly increased mortality. Perhaps most importantly, we observed that the 80–90% increase in mortality of immature bees exposed to captan was dose-independent and similar in magnitude to the lowest dose of thiamethoxam. This is an important result since it indicates captan is acutely toxic to honey bee larvae at field-realistic exposure levels. Furthermore, synergism between captan and thiamethoxam was observed to non-additively increase larval mortality at the highest dose of thiamethoxam, but there was no evidence of synergism or antagonism for other developmental stages or chemical dosages. At the full-colony level in the field, the effects of pesticide exposure at the lowest doses used in the laboratory assays did not translate to changes in colony performance as measured by bee population numbers, resistance to parasites/pathogens, or colony survivorship. Thus, we find that captan and thiamethoxam are each acutely toxic to immature honey bees, but whole colonies can potentially compensate for detrimental effects, at least at the low doses and duration used for our field trial, or that methodological differences of the field experiment impacted results (e.g., dilution of pollen patty treatments with uncontaminated natural pollen despite our season-long use of pollen traps on the colonies to minimize this effect).

Fungicides are not intended to control insects, and their acute toxicity to honey bees is generally low^49^. That said, a recent review shows that risk from some fungicide exposures can be high for bees^38^. Impacts of fungicides on honey bee brood have been observed^39,50,51^, which is in agreement with our findings regarding captan’s acute toxicity to developing larvae/pupae. Captan inhibits respiratory pathways in fungal cells, but this mode of action may also be detrimental to animals such as honey bees since they use similar metabolic pathways^41,52^. The larval stages of honey bees may be particularly susceptible to captan due to the high rate of mitochondrial biogenesis and respiratory metabolism in response to the large ATP demand at this stage of development^53,54^. Our results revealing acute toxicity of captan to honey bee larvae may be of interest to regulatory agencies such the United States Environmental Protection Agency (EPA) as a scientific line of evidence of this fungicide’s toxicity to immature honey bees. Reassessment of captan for its toxicity to honey bee larvae during the pesticide registration review process is currently recommended by the EPA^55^.

In addition to the direct acute toxicity of captan by itself, we also addressed the potential interaction of this fungicide with the neonicotinoid insecticide thiamethoxam. The combination of captan with thiamethoxam at the highest concentration tested did synergistically increase mortality of larvae. However, synergisms at other concentrations and at other developmental stages were not observed. Thiamethoxam is an insecticide distinguished for its lethality to bees in both immature and adult stages^30,35,56,57^. Despite this neonicotinoid impacting neurologic^27,31,32,58^ and immune systems^59,60^, the honey bee detoxification system does have some mechanisms to metabolize nicotine-like compounds^49,61^. Sterol biosynthesis-inhibiting (SBI) fungicides are known to interfere with these detoxification mechanisms^62^, but captan does not have the same mode of action as SBI fungicides and therefore the mechanism underlying synergism is unclear. Due to their limited number of detoxification genes compared to other insects, honey bees may have difficulty processing multiple xenobiotics simultaneously^61,63^.

Despite the findings of the in vitro assays, our field trial with full colonies did not observe impacts of pesticide exposure over one year at the low concentrations used in the study. The incongruity among lab and field toxicology studies of bees is important to consider. Carreck and Ratnieks^64^ list three main factors that can lead to incongruence between laboratory-based and field-based toxicology studies: pesticide concentration, duration of exposure, and the capacity of foragers to choose between foraging sites in the field. Our choice of pesticide concentrations for the in vitro lab assays (100, 500, and 2000 ppb for captan, and 10, 70, and 1,440 ppb for thiamethoxam) were based on a careful search of pesticide residues found in field surveys of honey bee-collected pollen and beebread (see Tables S7 & S8) and the concentrations chosen provide a range of field-realistic exposure possibilities. In addition, in the in vitro lab assay, larvae were exposed to pesticide treatments via diet from the fourth day of larval development (i.e., the transition from the fourth to the fifth larval instar, when worker larvae switch from an exclusive royal jelly diet to a mix of jelly with pollen and nectar^65^ until reaching the pupal stage). This protocol is what is advised by the Organization for Economic Cooperation and Development guidelines for toxicological studies (OECDTest No. 239^66^), indicating our laboratory bioassays were appropriate. Thus, our lab-based study was sound, and the field-based study shows that full colonies likely have mechanisms to compensate for increased larval mortality, or that methodological differences between the lab assay and field study contribute to differences in results. For example, even though we restricted natural pollen acquisition by foragers by placing pollen traps on the colonies, some natural pollen was acquired, thereby diluting the pesticide treatments in our pollen patties. Alternatively, if the pesticides did impact larval mortality in the field experiment, the queen may compensate for increased larval mortality by laying more eggs. Short-term colony resilience has been described among colonies exposed to sub-lethal dosages of neonicotinoids, whereby colonies increase brood initiation rate to compensate for increased brood mortality^67,68^. While effective in the short term, this could lead to longer-term costs for the queen and an increased incidence of queen failure^69,70^.

The degree to which nurse bees in honey bee colonies can detoxify xenobiotic substances in pollen before secreting jelly to the brood is poorly known, but the limited evidence to date suggests this can be part of an effective “social detoxification system”^61,71,72^. For example, Ricke et al.^73^ found that less than 2% of pesticides in pollen fed to nurse bees made it into jelly excreted by the workers. Moreover, royal jelly from colonies exposed to pesticide-contaminated pollen contained negligible residues, although queens reared with this jelly had reduced reproductive quality^74^. It is also worth considering that while nurse bees may remove some residues from jelly fed to larvae, the larvae may also be exposed to pesticides via other hive matrices, such as wax^75,76^. At immature stages, exposure occurs transdermally and orally at the same time, and larvae interact extensively with the wax comb in their cells. This may be especially important given that early life stages of bees are generally more sensitive to contaminants relative to adult stages^13^.

Overall, our laboratory and field results show that field-realistic exposures to the fungicide captan greatly increase immature honey bee mortality, and synergism with the neonicotinoid thiamethoxam occurs, but synergism is dose-dependent and full colonies may be able to compensate for losses. Therefore, we suggest that further effort be placed in understanding how exposure to captan and co-exposure with other pesticides affects honey bee colony dynamics and mortality of solitary and sub-social wild bees, which lack the social detoxification capabilities of honey bees and can therefore be more susceptible to pesticides in the field. Notably, captan affected honey bee larval survivorship regardless of the concentration and at field-realistic levels. Most studies investigating captan describe effects after chronic exposure^39,41,51,77,78^, while our study found an impact via a single exposure event. Since captan is actively applied to crops during bloom while honey bees and wild pollinators are carrying out critical pollination services, further investigations of this fungicide are warranted to ensure the sustainability of crop pollination and agricultural production.

## Methods

### Chemicals

The agrochemicals used for toxicity assays were the fungicide captan (CAS number 133-06-2, ≥ 98% purity); and the insecticide thiamethoxam (CAS number 153719-23-4, ≥ 98% purity), both from Sigma-Aldrich. The fungicide captan is one of the most-used fungicides in the United States and is the most-sprayed fungicide during apple bloom in NY^18^. Thiamethoxam is a widely used neonicotinoid insecticide^28^ and has been identified as one of the highest-risk pesticides to honey bees worldwide^24^.

### In vitro laboratory assays

We used an in vitro rearing method to assess the impact of pesticide exposure on individual honey bees during development. Worker larvae were sampled from six honey bee colonies (stock from Chuck Kutik, Kutik’s Honey Bees, which are not selected for any specific traits such as varroa resistance) located at the Dyce Lab for Honey Bee Studies at Cornell University in Ithaca, New York. Worker larvae at the first instar were grafted individually in commercial plastic queen cups, which were placed inside 96-well microcentrifuge tube racks containing equal numbers of wells filled with a saturated K_2_SO_4_, and using a feeding protocol previously described for honey bee worker rearing in an artificial environment^66,79^ with slight modifications on the volume of liquid diet provided (Table 2). The larvae were treated with diet administered on day 4, with measurement of effects during the larval and pupal phases up through adult emergence (daily observations between days 5-22).

**Table 2:** Larval feeding regimen used in the in vitro rearing system.

Larval progress	Diet	Amount of diet (μl)
Day 0 (at grafting)	A	30
Day 1	N/A	0
Day 2	B	30
Day 3	C	40
Day 4	C	50
Day 5	C	60

The diet provided were A (44.25% of royal jelly, 5.3% of D-Glucose, 5.3% of D-Fructose, 0.9% of Yeast extract and 44.25 distilled water), B (42.94% of royal jelly, 6.4% of D-Glucose, 6.4% of D-Fructose, 1.3% of Yeast extract and 42.95 distilled water) or C (50% of royal jelly, 9% of D-Glucose, 9% of D-Fructose, 2% of Yeast extract and 30% distilled water).

Glucose (CAS Number: 50-99-7) and Fructose (CAS Number: 57-48-7) were obtained from Sigma-Aldrich (St. Louis, MO, USA), yeast extract (#288620) was obtained from Life Technologies Corp. (Sparks, MD, USA) and organic royal jelly was obtained from Glorybee (Eugene, OR, USA). Diets were prepared and provided in different amounts according to each larval stage. The larvae were kept in an incubator at 34.5 °C and 80% RH until the defecation stage, then at 70% RH during the pupal stage until emergence of the adults^80^.

Under normal development, worker larvae switch from an exclusive royal jelly diet to a mix of jelly with pollen and nectar between the fourth to the fifth larval instar^65^. Thus, we applied the pesticide treatments at this phase, following the OECD 239 study design^66^. Cautious attention was taken to select only larvae that had achieved the fourth larval instar^81^, thus ensuring the next stages of developmental monitoring were accurate. Treatment solutions were mixed into the diet and supplied individually to larvae according to each treatment group. The concentrations used were based on sublethal concentrations previously found in the literature (see Tables S7 and S8). For the insecticide thiamethoxam, the concentrations were: low (10 ng/ml = 10 parts per billion; *n* = 94 larval replicates), medium (70 ng/ul; *n* = 95) and high (1440 ng/ml; *n* = 94). For the fungicide captan, the concentrations were: low (100 ng/ml; *n* = 92), medium (500 ng/ml; *n* = 96) and high (2000 ng/ml; *n* = 96). We tested for potential interactions between these pesticides by using a blend of captan at high concentration (2000 ng/ml) and thiamethoxam at low (10 ng/ml; *n* = 109), medium (70 ng/ul; *n* = 109) and high (1440 ng/ml; *n* = 103) concentrations. Both chemicals were solubilized in acetone before combining with the jelly diet. Positive (+ acetone; *n* = 236) and negative (no acetone; *n* = 235) control groups were tested to measure possible effects of the solvent. Three experimental trials were conducted at different times; see Table S1 for the number of treated larvae in each treatment group and trial. The cups containing the diet with different treatments were randomly distributed in the racks to avoid site effects and controls were included in every rack.

All larvae were monitored twice a day and larvae that died (movement stopped, discoloring, drowned in the food) or if there was any evidence of contamination, were immediately recorded and removed. Survivorship and developmental advances were recorded during all ontogenetic stages until each larva reached adulthood. Adults were considered viable if they successfully crossed the pupation stages and reached adulthood healthy, morphologically well-shaped, and actively walking around.

### Field trials with whole colonies

Forty-eight nucleus honey bee colonies were acquired from a commercial beekeeper (Chuck Kutik, Norwich, NY). All nucleus colonies were transferred into a new 10-frame box with plastic foundation and allowed to draw comb for two weeks in the same location (the Dyce Lab for Honey Bee Studies, Ithaca, NY, USA). After this period, the colonies were distributed to three local apiary locations as described below, with equal representation of treatments at each apiary. Before starting the experiments, all colonies were checked for queen status, number of bees, and frame composition. Frames were redistributed among colonies such that all colonies had a similar composition of brood, bees, pollen and nectar immediately prior to enrollment in the field experiment. In addition, each colony was fitted with a pollen trap, placed at the entrance of the hive, to restrict pollen flow into the hive and therefore ensure consumption of the treated pollen patties^67^.

Twelve colonies were assigned to each of four treatments: control (no pesticides added), thiamethoxam (10 ppb), captan (1000 ppb), and thiamethoxam + captan (10 ppb thiamethoxam + 1000 ppb captan). We used the lowest dose of thiamethoxam from our laboratory assays (10 ppb) and a medium/high dose of captan (1000 ppb) because these contamination levels are commonly observed from our own studies during crop pollination^17,18^ and the broader literature (see Tables S7 and S8). Pollen patty treatments were prepared the day before they were fed to the bees using captan (CAS number 133-06-2, ≥ 98% purity) and thiamethoxam (CAS number 153719-3-4, ≥ 98% purity), both from Sigma-Aldrich (St. Louis, MO, USA), and Bee Pro from Mann Lake (Hackensack, MN, USA). The pollen patties were provided to each colony weekly from the first week of June to the first week in October 2016, in accordance with some local beekeeping practices. Patties were kept on for 3–7 days, with an average of 4 days, until they were almost entirely consumed. Two hives had patties left on for 14 days in one month. Because the colonies accumulated minimal honey during the summer, likely due to the fact that pollen traps were on the colonies continuously from the start of the experiment to ensure consumption of the treated patties^67^, we supplementally fed each colony three liters of sugar syrup (30% by volume) per week from the third week of August until the first week first week of October. We made this decision because all of the colonies would likely have died over the winter from insufficient honey stores. Sugar syrup was prepared weekly according to treatments and thus represented an additional dietary implementation of our treatments: thiamethoxam (10 ppb), captan (1000 ppb), and thiamethoxam + captan (10 ppb thiamethoxam + 1,000 ppb captan). The colonies were equally distributed among three apiaries: Varna (42.463730, − 76.440748), Turkey Hill (42.437293, − 76.427063), and Sarkaria (42.443964, − 76.452238); which are ~ 2 km from each other. The colonies were not treated for varroa mites during the experiment, and overwintering preparation consisted of placing a moisture board and foam-insulated inner cover under the telescoping outer cover.

Performance and survival of colonies were assessed via monthly inspections in June, July, August, October, and November 2016, and March, April, May, and June 2017. During each June-October 2016 and April 2017 visit, we estimated the number of adult bees, brood cells, nectar cells, and pollen cells of each surviving colony. We followed the Liebefeld method^82^, visually estimating the proportion of each frame covered by adult bees or each type of cell and summing these proportions across all frames in a hive. Additionally, each colony had one drone comb frame, and the proportion of the frame covered by drone brood was recorded once per month in July, August, September, October 2016 and April 2017. The inspections also accounted for queen supersedure events, varroa levels (measured as mites per 300 bees using the sugar roll method) and chalkbrood presence. Colony survival was monitored during all 2016 and 2017 visits.

### Statistical analyses

#### In vitro laboratory assays

We assessed the impact of treatment on the survival of immature bees using a Cox proportional hazards model for survivorship, as well as a logistic model for the percentage mortality at the end of the trial (Fig. 2). In each model, both the pesticide treatment and trial number were included as additive fixed effects, the latter to account for variation in natural mortality between trials. Post-hoc tests for pairwise differences between treatments, marginalized across trials, were performed for both models, adjusting for multiple comparisons using Tukey’s HSD.

We used Bliss’ definition of independence to define synergism or antagonism between different pesticides^83^. Although Bliss independence is traditionally defined using mortality rates, it can also be defined using survival rates. Let ${s}_{0}$ be the control survival rate, ${s}_{0}{s}_{\text{A}}$ the survival rate when exposed to pesticide A, and ${s}_{0}{s}_{\text{B}}$ the survival rate when exposed to pesticide B; here ${s}_{\text{A}}$ and ${s}_{\text{B}}$ can be interpreted as Abbott-corrected survival rates^84^. If pesticides A and B are Bliss-independent, then the survival rate when simultaneously exposed to both pesticides is given by ${s}_{0}{s}_{\text{A}}{s}_{\text{B}}$; if the actual survival rate is lower, then the two pesticides interact synergistically, whereas if the reverse is true, then they interact antagonistically. Biologically, Bliss independence requires that the two pesticides have independent biological action, and also that individual susceptibilities to A and to B are uncorrelated. The second requirement is important but often overlooked: as an extreme example, if the susceptibilities were perfectly correlated, then all bees that survive pesticide A will also survive any additional exposure to pesticide B, so the survival rate remains at ${{s}_{0}{s}_{\text{A}}<s}_{0}{s}_{\text{A}}{s}_{\text{B}}$, leading to an apparent antagonism.

We developed a new approach to assess Bliss independence between pesticides A and B, based on the well-established framework of the generalized linear model (GLM). The idea is as follows. We fitted *survivorship* using a binomial GLM with a *log* link function (as opposed to typical binomial GLMs for *mortality* with *logit* link functions), with binary (0/1) predictors ${x}_{\text{A}}$ and ${x}_{\text{B}}$, representing exposure to pesticide A and to B, as well as the interaction ${x}_{\text{A}}{x}_{\text{B}}$. The log link function means that

$$\text{log}\left({p}_{\text{surv}}\right)={\beta }_{0}+{\beta }_{\text{A}}{x}_{\text{A}}+{\beta }_{\text{B}}{x}_{\text{B}}+{\beta }_{\text{AB}}{x}_{\text{A}}{x}_{\text{B}},$$

so the survival rates are

$${p}_{\text{surv}}=\left\{\begin{array}{ll}\text{exp}\left({\beta }_{0}\right),& \text{control,}\\ \text{exp}\left({\beta }_{0}\right)\text{exp}\left({\beta }_{\text{A}}\right),& \text{exposed to pesticide A only,}\\ \text{exp}\left({\beta }_{0}\right)\text{exp}\left({\beta }_{\text{B}}\right),& \text{exposed to pesticide B only,}\\ \text{exp}\left({\beta }_{0}\right)\text{exp}\left({\beta }_{\text{A}}\right)\text{exp}\left({\beta }_{\text{B}}\right)\text{exp}\left({\beta }_{\text{AB}}\right),& \text{simultaneous exposure to A and B.}\end{array}\right.$$

If pesticides A and B are Bliss-independent, then the survival rate for simultaneous exposure should simply be

$${p}_{\text{surv}}=\text{exp}\left({\beta }_{0}\right)\text{exp}\left({\beta }_{\text{A}}\right)\text{exp}\left({\beta }_{\text{B}}\right),$$

so we require that $\text{exp}\left({\beta }_{\text{AB}}\right)=1$, or equivalently ${\beta }_{\text{AB}}=0$. Therefore, Bliss independence is equivalent to the null hypothesis that the interaction ${x}_{\text{A}}{x}_{\text{B}}$ has no effect, which can be evaluated using a likelihood ratio test. A significant negative ${\beta }_{\text{AB}}$ would imply synergism, and a significant positive ${\beta }_{\text{AB}}$ would imply antagonism. The use of a log-binomial GLM also allows us to include the trial number as an additional fixed effect to account for variations in natural mortality between trials. Hence our method improves upon the method in^85^, not only by accounting for natural mortality, but also by allowing for variations between experimental replicates. We tested for interactions between the fungicide and the insecticide at three different chemical dosages on the overall survivorship, as well as the survivorship within each of three benchmarks of ontogenic development (5^th^ larval instar, Pre-pupae and pupation). Holm correction was used to account for multiple testing.

All analyses and graphical presentation were conducted in R version 3.5.1^86^, using the packages "survival"^87^ and “survminer”^88^ for the analysis of survival curves, "emmeans"^89^ for post-hoc pairwise tests between treatments, and “lbreg”^90^ for fitting log-binomial models in the Bliss independence analysis.

### Field trials with whole colonies

First, we tested whether pollen patty consumption (grams consumed per day) varied across treatments. Treatment, month, the treatment × month interaction, and apiary location were fixed effects, and colony identity was a random effect. We also included the estimated number of adult bees during performance assessments, as a covariate. Month was treated as a categorical predictor because the number of adult bees was not measured in September; we estimated this value by averaging the number of adult bees in October and August. Significance of model terms here and below was assessed with likelihood ratio tests. We used post-hoc Tukey pairwise comparisons to test for differences in pollen patty consumption between months.

Regarding the survival analysis, we performed a Kaplan–Meier survival curves for each treatment; *surv function*, ‘survival’ package^87^. We assessed statistical differences in survival among treatments with a log-rank test, using treatment as the main effect and apiary location as a frailty term (*survdiff function*).

To examine the performance ratios, we used separate linear mixed models to test how treatment influenced the frame area of adult bees, nectar cells, pollen cells, and worker brood cells. Treatment, month, the treatment × month interaction, and apiary location were fixed effects, and colony identity was a random effect. We also included the frame area of adult bees as a covariate when modeling the frame area of brood cells, nectar cells, and pollen cells. The proportion of the drone brood frame covered by drone brood was modeled with the same fixed and random effects using a generalized linear mixed model and a Tweedie error distribution; glmmTMB function, ‘TMB’ package^91^. When month was significant in any of the above performance metrics, we used post-hoc Tukey pairwise comparisons to test for differences between months.

The possible event of supersedure of the original queen (yes/no) at any point in the study was modeled as a function of treatment and apiary location using penalized logistic regression due to small number of positive cases (logistf function, ‘logistf’ package^92^. For parasites and pathogens, we assessed varroa levels (mites per 300 bees, log(x + 1) transformed) with a linear mixed model. Treatment, month, the treatment × month interaction, and site (apiary locations) were included as fixed effects, and colony identity was included as a random effect. We modeled the presence of chalkbrood (yes/no) at any point in the study as a function of treatment and apiary location using logistic regression.

### Supplementary Information

Supplementary Information.

## Data Availability

Data is provided within the manuscript or supplementary information files.
