# Supplementary material for: Acute toxicity of the fungicide captan to honey bees and mixed evidence for synergism with the insecticide thiamethoxam
Source: Sci Rep. 2024 Jul 8;14:15709. doi: 10.1038/s41598-024-66248-x (PMC11231156; doi:10.1038/s41598-024-66248-x)
Supplement: Supplementary file 1 — Supplementary Information. [file 41598_2024_66248_MOESM1_ESM.docx]

**Table S1**. Starting number of larvae (on Day 4, when the treatment was applied) for each treatment and experimental trial of the in-vitro assays.

|  | **Trial 1** | **Trial 2** | **Trial 3** |
| --- | --- | --- | --- |
| **Control (-)** | 61 | 94 | 80 |
| **Control (+)** | 61 | 95 | 80 |
| **Captan low** | 16 | 32 | 44 |
| **Captan med** | 16 | 32 | 48 |
| **Captan high** | 16 | 32 | 48 |
| **Thiamethoxam low** | 16 | 32 | 46 |
| **Thiamethoxam med** | 16 | 32 | 47 |
| **Thiamethoxam high** | 16 | 32 | 46 |
| **Cap. high + Thia. low** | 29 | 32 | 48 |
| **Cap. high + Thia. med** | 29 | 32 | 48 |
| **Cap. high + Thia. high** | 29 | 26 | 48 |

**Table S2**. Pairwise comparisons for the effects of treatment on the proportional hazards in the in-vitro assays, from the Cox model. Estimates are for column label minus row label; for example, the upper left cell indicates that the hazard rate for “Control (-)” is exp(0.243)=1.28 times that of “Control (+)”. Tukey corrections have been applied to the p-values. Significant negative estimates (higher survival for column label) are shown in red. No significant positive estimates were found.

|  | **Control (-)** | **Control (+)** | **Cap. low** | **Cap. med** | **Cap. high** | **Thia. low** | **Thia. med** | **Thia. high** | **C high + T low** | **C high + T med** |
| --- | --- | --- | --- | --- | --- | --- | --- | --- | --- | --- |
| **Control (+)** | est = 0.243, p = 0.949 |  |  |  |  |  |  |  |  |  |
| **Cap. low** | est = -0.565, p = 0.105 | est = -0.808, p = 0.002 |  |  |  |  |  |  |  |  |
| **Cap. med** | est = -0.47, p = 0.351 | est = -0.713, p = 0.017 | est = 0.095, p = 1.000 |  |  |  |  |  |  |  |
| **Cap. high** | est = -0.53, p = 0.186 | est = -0.773, p = 0.006 | est = 0.035, p = 1.000 | est = -0.06, p = 1.000 |  |  |  |  |  |  |
| **Thia. low** | est = -0.712, p = 0.008 | est = -0.955, p = < 0.001 | est = -0.146, p = 1.000 | est = -0.241, p = 0.989 | est = -0.182, p = 0.999 |  |  |  |  |  |
| **Thia. med** | est = -0.841, p = < 0.001 | est = -1.083, p = < 0.001 | est = -0.275, p = 0.965 | est = -0.37, p = 0.805 | est = -0.31, p = 0.928 | est = -0.129, p = 1.000 |  |  |  |  |
| **Thia. high** | est = -0.987, p = < 0.001 | est = -1.23, p = < 0.001 | est = -0.422, p = 0.584 | est = -0.517, p = 0.287 | est = -0.457, p = 0.478 | est = -0.275, p = 0.953 | est = -0.147, p = 1.000 |  |  |  |
| **C high + T low** | est = -0.964, p = < 0.001 | est = -1.206, p = < 0.001 | est = -0.398, p = 0.645 | est = -0.493, p = 0.336 | est = -0.433, p = 0.540 | est = -0.252, p = 0.971 | est = -0.123,  p = 1.000 | est = 0.023, p = 1.000 |  |  |
| **C high + T med** | est = -1.031, p = < 0.001 | est = -1.274, p = < 0.001 | est = -0.465, p = 0.395 | est = -0.56, p = 0.159 | est = -0.501, p = 0.305 | est = -0.319, p = 0.864 | est = -0.19, p = 0.996 | est = -0.044, p = 1.000 | est = -0.067, p = 1.000 |  |
| **C high + T high** | est = -1.624, p = < 0.001 | est = -1.867, p = < 0.001 | est = -1.058, p = < 0.001 | est = -1.153, p = < 0.001 | est = -1.094, p = < 0.001 | est = -0.912, p = < 0.001 | est = -0.783, p = 0.001 | est = -0.637, p = 0.015 | est = -0.660, p = 0.006 | est = -0.593, p = 0.022 |

**Table S3**. Pairwise comparisons for the effects of treatment on the percentage mortality at the end of the in-vitro assays, from the logistic model. Estimates are for column label minus row label; for example, the upper left cell indicates that logit(mortality) for “Control (-)” is larger than that of “Control (+)” by 0.257. Tukey corrections have been applied to the p-values. Significant negative estimates (higher survival for column label) are shown in red. No significant positive estimates were found.

|  | **Control (-)** | **Control (+)** | **Cap. low** | **Cap. med** | **Cap. high** | **Thia. low** | **Thia. med** | **Thia. high** | **C + T low** | **C + T med** |
| --- | --- | --- | --- | --- | --- | --- | --- | --- | --- | --- |
| **Control (+)** | est = 0.267, p = 0.972 |  |  |  |  |  |  |  |  |  |
| **Cap. low** | est = -0.840, p = 0.045 | est = -1.107, p = 0.001 |  |  |  |  |  |  |  |  |
| **Cap. med** | est = -0.687, p = 0.208 | est = -0.954, p = 0.011 | est = 0.154, p = 1.000 |  |  |  |  |  |  |  |
| **Cap. high** | est = -0.687, p = 0.208 | est = -0.954, p = 0.011 | est = 0.154, p = 1.000 | est = 0.000, p = 1.000 |  |  |  |  |  |  |
| **Thia. low** | est = -0.896, p = 0.021 | est = -1.163, p = < 0.001 | est = -0.056, p = 1.000 | est = -0.21, p = 1.000 | est = -0.21, p = 1.000 |  |  |  |  |  |
| **Thia. med** | est = -1.012, p = 0.004 | est = -1.279, p = < 0.001 | est = -0.172, p = 1.000 | est = -0.325, p = 0.992 | est = -0.325, p = 0.992 | est = -0.115, p = 1.000 |  |  |  |  |
| **Thia. high** | est = -1.347, p = < 0.001 | est = -1.614, p = < 0.001 | est = -0.507, p = 0.85 | est = -0.661, p = 0.509 | est = -0.661, p = 0.509 | est = -0.451, p = 0.922 | est = -0.335, p = 0.990 |  |  |  |
| **C + T low** | est = -1.181, p = < 0.001 | est = -1.448, p = < 0.001 | est = -0.341, p = 0.986 | est = -0.494, p = 0.830 | est = -0.494, p = 0.830 | est = -0.284, p = 0.996 | est = -0.169, p = 1.000 | est = 0.166, p = 1.000 |  |  |
| **C + T med** | est = -1.259, p = < 0.001 | est = -1.526, p = < 0.001 | est = -0.418, p = 0.94 | est = -0.572, p = 0.665 | est = -0.572, p = 0.665 | est = -0.362, p = 0.977 | est = -0.247, p = 0.999 | est = 0.088, p = 1.000 | est = -0.078, p = 1.000 |  |
| **C + T high** | est = -2.035, p = < 0.001 | est = -2.302, p = < 0.001 | est = -1.195, p = 0.006 | est = -1.349, p = < 0.001 | est = -1.349, p = < 0.001 | est = -1.139, p = 0.011 | est = -1.023, p = 0.039 | est = -0.688, p = 0.507 | est = -0.855, p = 0.144 | est = -0.777, p = 0.262 |

**Table S4**. Likelihood ratio tests for Bliss interaction between thiamethoxam (three dosages) and captan (high dosage) in the in-vitro assays, based on log-binomial GLMs of survivorship. Since Bliss interaction is traditionally defined using mortality, we have converted survivorships estimated from the GLMs into the Abbott-corrected mortalities shown below. The larval data included all starting larvae, the pre-pupal data only included larvae that had survived to the pre-pupa stage, and the pupal data only included those that had further survived to the pupa stage. Two sets of Holm adjustments were made, one for the analyses of overall survivorship (3 tests), and one for the analyses of survivorship within each developmental stage (9 tests).

| **Dev. stage** | **Thiamat. dosage** | **Mortality (Captan)** | **Mortality (Thiamat.)** | **Mortality (Combined)** | **Interaction** | **χ^2^**(1) | **p-value (Holm)** |
| --- | --- | --- | --- | --- | --- | --- | --- |
| All stages combined (overall survivorship) | Low | 0.241 | 0.298 | 0.401 | Antagonism | 0.461 | 1.000 |
|  | Medium | 0.240 | 0.341 | 0.411 | Antagonism | 0.834 | 1.000 |
|  | High | 0.243 | 0.461 | 0.644 | Synergism | 0.359 | 1.000 |
| Larva | Low | 0.080 | 0.010 | 0.165 | Synergism | 1.218 | 1.000 |
|  | Medium | 0.075 | 0.041 | 0.191 | Synergism | 1.200 | 1.000 |
|  | High | 0.074 | 0.014 | 0.299 | Synergism | 7.932 | 0.044 |
| Pre-pupa | Low | 0.062 | 0.177 | 0.096 | Antagonism | 3.153 | 0.531 |
|  | Medium | 0.062 | 0.219 | 0.085 | Antagonism | 5.984 | 0.115 |
|  | High | 0.063 | 0.234 | 0.233 | Antagonism | 0.333 | 1.000 |
| Pupa | Low | 0.098 | 0.113 | 0.199 | Antagonism | 0.000 | 1.000 |
|  | Medium | 0.089 | 0.035 | 0.191 | Synergism | 0.430 | 1.000 |
|  | High | 0.099 | 0.212 | 0.302 | Synergism | 0.010 | 1.000 |

**Table S5:** **Likelihood ratio test results for model of pollen patty consumption.** Pollen patties were treated with captan, thiamethoxam, captan and thiamethoxam combined, or left untreated (control). Pollen patties were placed in hives approximately once a week from June to October.

| **Source** | **Degrees of freedom** | | **χ^2^** | ***p*** |
| --- | --- | --- | --- | --- |
| Treatment | | 3 | 1.54 | 0.67 |
| Month | | 4 | 398.51 | <0.0001 |
| Area of adult bees | | 1 | 42.83 | <0.0001 |
| Bee yard | | 2 | 0.64 | 0.72 |
| Treatment × month | | 12 | 18.58 | 0.099 |

**Table S6**. Likelihood ratio test results for separate models to test the effect of pesticide treatments on *Varroa* levels in the field trials.

| **Source** | **Degrees of freedom** | **χ^2^** | **p** |
| --- | --- | --- | --- |
| Treatment | 3 | 2.23 | 0.53 |
| Month | 2 | 51.86 | <0.0001 |
| Bee yard | 2 | 0.48 | 0.79 |
| Treatment × month | 6 | 8.09 | 0.23 |

**Table S7:** **Thiamethoxam Surveys.**

| **Paper** | **Residue** |
| --- | --- |
| (Mullin et al., 2010) | 53.0 ppb in beebread (mean of positives) |
| (Rennich et al., 2013) | 13.5 ppb in beebread (mean of positives) |
| (Krupke et al., 2012) | 1.2-7.4 ppb in trapped pollen (range of positives) |
| (Dively and Kamel, 2012) | 54.8-127.0 ppb in pollen of treated pumpkin plants (range of all) |
| (Pilling et al., 2013) | 1.0-7.0 ppb in trapped pollen (range of all) |
| (Sanchez-Bayo and Goka, 2014) | 28.9 ppb in beebread (mean of positives) 127.0 ppb (max) |
| (Botías et al., 2015) | ≤0.12-86.02 ppb in pollen of wildflowers in oilseed rape field margins (range of all) |
| (Long and Krupke, 2016) | 0.07-1.82 ppb in trapped pollen (range of positives) |
| (McArt et al., 2017) | 21.5 ppb in beebread (mean of positives) |
| (Frazier et al., 2015) | 12.7 ppb in trapped pollen (mean of all) |

**Table S8:** **Captan Surveys.**

| **Paper** | **Residue** |
| --- | --- |
| (Kubik et al., 2000) | 6,390 ppb in beebread (mean of all) |
| (Mullin et al., 2010) | 433.5 ppb in beebread (mean of all)  16.0-10000.0 ppb (range of positives) |
| (Bernal et al., 2010) | 17 ppb in beebread (mean of positives) |
| (Rennich et al., 2013) | 411 ppb in beebread (mean positives)  72.6–4900.0 ppb (range of positives) |
| (Pettis et al., 2013) | 976.9 ppb in trapped pollen (mean of all)  13800 ppb (max) |
| (Sanchez-Bayo and Goka, 2014) | 820.8 ppb in beebread (mean of positives)  10363 ppb (max) |
| (Frazier et al., 2015) | 1310.0 ppb in trapped pollen (mean of all from blueberry pollination)  51.5 ppb in trapped pollen (mean of all from apple pollination) |

**Figure S1:** Pollen patty consumption across four pesticide treatments applied from June to October 2016. Different letters indicate significant differences in pollen consumption between months (p < 0.05) according to post-hoc Tukey pairwise comparisons.

**Figure S2:** Percent drone comb frame covered by drone brood across four pesticide treatments applied from June to October 2016. Different letters below month names indicate significant differences between months (p < 0.05) according to post-hoc Tukey pairwise comparisons.

**Figure S3:** *Varroa* levels across four pesticide treatments applied from June to October 2016. Different letters below month names indicate significant differences in *Varroa* levels between months (p < 0.05) according to post-hoc Tukey pairwise comparisons.

Figure S1:


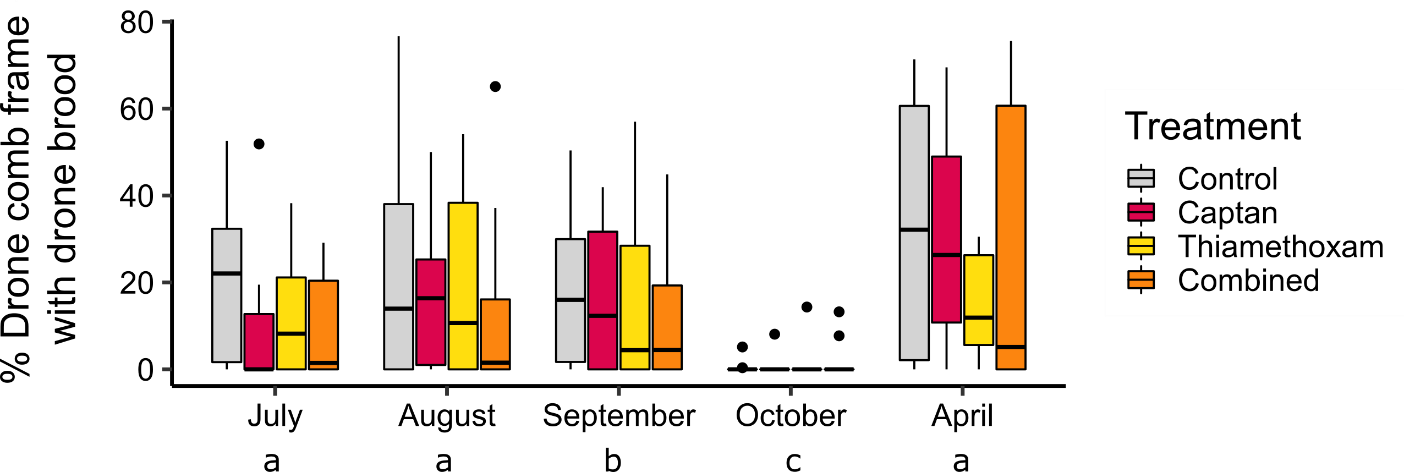


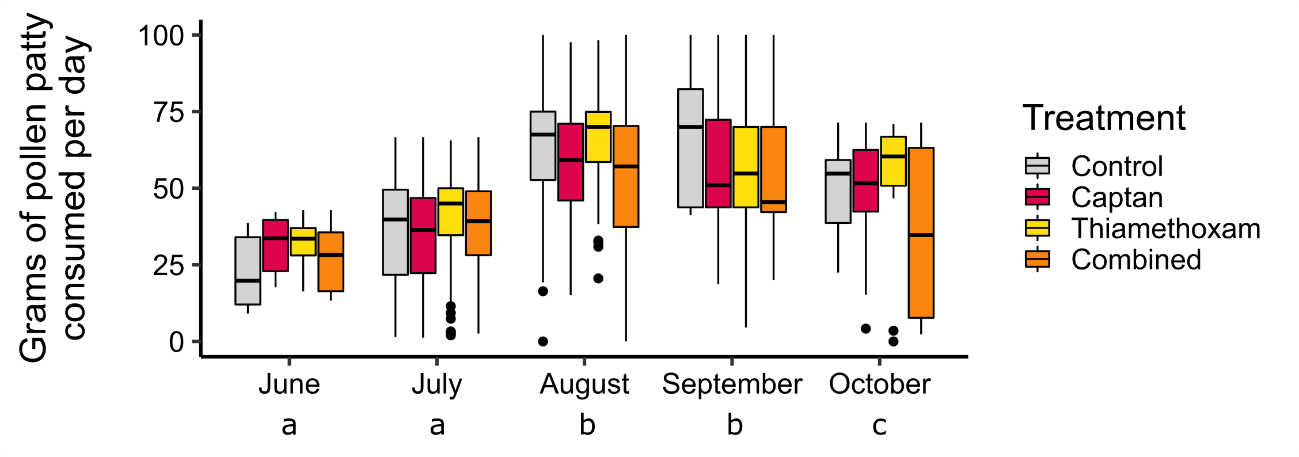
Figure S2:

Figure S3


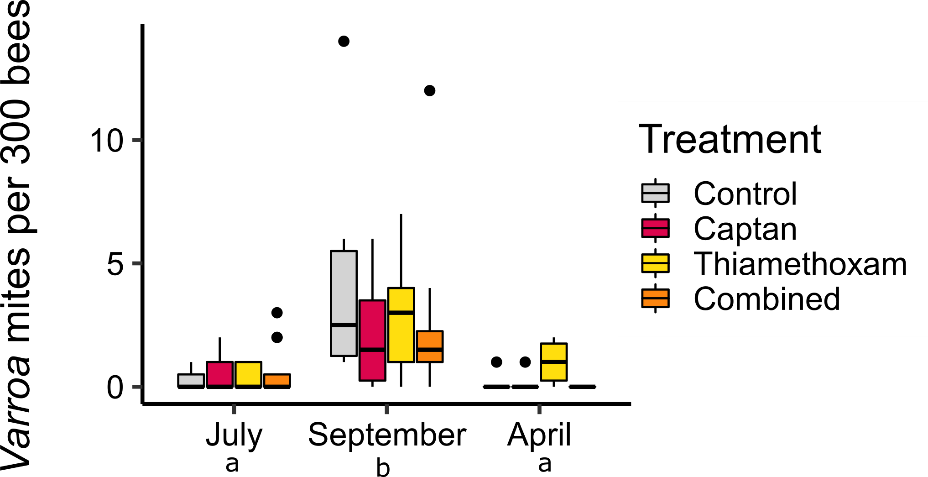


**References:**

Bernal, J., Garrido-Bailón, E., Del Nozal, M. J., González-Porto, A. V., Martín-Hernández, R., Diego, J. C., Jiménez, J. J., Bernal, J. L., and Higes, M. (2010). Overview of pesticide residues in stored pollen and their potential effect on bee colony (Apis mellifera) losses in Spain. *J Econ Entomol* **103**, 1964-71.

Botías, C., David, A., Horwood, J., Abdul-Sada, A., Nicholls, E., Hill, E., and Goulson, D. (2015). Neonicotinoid Residues in Wildflowers, a Potential Route of Chronic Exposure for Bees. *Environ Sci Technol* **49**, 12731-40.

Dively, G. P., and Kamel, A. (2012). Insecticide residues in pollen and nectar of a cucurbit crop and their potential exposure to pollinators. *J Agric Food Chem* **60**, 4449-56.

Frazier, M., Mullin, C., Frazier, J., Ashcraft, S., Leslie, T., Mussen, E., and Drummond, F. (2015). Assessing Honey Bee (Hymenoptera: Apidae) Foraging Populations and the Potential Impact of Pesticides on Eight U.S. Crops. *Journal of Economic Entomology* **108**, tov195.

Krupke, C. H., Hunt, G. J., Eitzer, B. D., Andino, G., and Given, K. (2012). Multiple Routes of Pesticide Exposure for Honey Bees Living Near Agricultural Fields. *PLOS ONE* **7**, e29268.

Kubik, M., Nowacki, J., Pidek, A., Warakomska, Z., Michalczuk, L., Goszczyñski, W., and Dwużpnik, B. (2000). Residues of captan (contact) and difenoconazole (systemic) fungicides in bee products from an apple orchard. *Apidologie* **31**, 531-541.

Long, E., and Krupke, C. (2016). Non-cultivated plants present a season-long route of pesticide exposure for honey bees. *Nature Communications* **7**, 11629.

McArt, S. H., Fersch, A. A., Milano, N. J., Truitt, L. L., and Böröczky, K. (2017). High pesticide risk to honey bees despite low focal crop pollen collection during pollination of a mass blooming crop. *Scientific Reports* **7**, 46554.

Mullin, C. A., Frazier, M., Frazier, J. L., Ashcraft, S., Simonds, R., vanEngelsdorp, D., and Pettis, J. S. (2010). High Levels of Miticides and Agrochemicals in North American Apiaries: Implications for Honey Bee Health. *PLOS ONE* **5**, e9754.

Pettis, J., Lichtenberg, E., Andree, M., Stitzinger, J., Rose, R., and VanEngelsdorp, D. (2013). Crop Pollination Exposes Honey Bees to Pesticides Which Alters Their Susceptibility to the Gut Pathogen Nosema ceranae. *PloS one* **8**, e70182.

Pilling, E., Campbell, P., Coulson, M., Ruddle, N., and Tornier, I. (2013). A Four-Year Field Program Investigating Long-Term Effects of Repeated Exposure of Honey Bee Colonies to Flowering Crops Treated with Thiamethoxam. *PLOS ONE* **8**, e77193.

Rennich, K., Pettis, J., VanEngelsdorp, D., Bozarth, R., Eversole, H., Roccasecca, K., Smith, M., Stitzinger, J., Andree, M., Snyder, R., Rice, N., Evans, J., Levi, V., Lopez, D., and Rose, R. (2013). 2011-2012 National honey bee pests and diseases survey report*. pp. 53-68.

Sanchez-Bayo, F., and Goka, K. (2014). Pesticide Residues and Bees – A Risk Assessment. *PLOS ONE* **9**, e94482.
